# Use of HDPE implants in facial skeletal augmentation: Should we rush for it?

**Published:** 2010

**Authors:** Ramesh Kumar Sharma

**Affiliations:** Department of Plastic Surgery, PGIMER, Chandigarh-160 012, India

This article deals with the use of HDPE implant in the facial skeletal augmentation. The HDPE implants were used to augment the nasal dorsum, maxilla, malar eminence, chin, mandible body and angle, orbital rim and frontal region. The authors claim that majority of the patients had "satisfactory" results and the complication rate was about 10%. In this series none of the patients required implant removal till the time of reporting in their series. Let us dwell into the details of the 'satisfactory results'. Who has analyzed the patients' photographs—the surgeon or some third person? What criteria were taken to document improvement in the patient contour/appearance? Has the observation been put to some statistical scrutiny? In the absence of any objective criteria, the claims of improvement tend to become anecdotal.

What we do notice, however, is that the complication rate in the "nasal subgroup" is rather high (21% complications and 7% exposure). The authors feel that the complications could be avoided by having a slightly undersized implant and by ensuring a minimally scarred pocket. Many researchers have concluded that placement of implants directly under the skin without coverage of periosteum or another fascial envelope has increased risk of early and late exposure. Morbidity is also dependent upon the route chosen for implant replacement. The transoral and transnasal routes lead to more chances of infection.[1]

We are a proponent of autogenous material for correction of contour deformities. In a large personal series of more than 80 cases over a period of last 15 years there has not been even a single case where the autogenous material got infected or had to be removed later on. There is always a lurking danger of implant exposure even on a trivial trauma. Unfortunately, contrary to the claim of being able to "salvage" the implant by trimming and covering with vascularized tissue,[2,3] in practice, the exposed implant needs to be removed, and one has to resort to substituting it with the good old and reliable autogenous material. One must also keep in mind that in the event of implant necessitating removal, it would be a surgical nightmare to do so for the HDPE implant that is badly stuck in the fibrous in-growth. When erosions occur they spoil the overlying skin and even after implant removal, the overlying tissues may be ruined for ever.[4] The HDPE implants are not far from being inert from an immunological point of view. Nevertheless, the reaction does not seem to be severe enough to compromise the stability and volume of the implant from a clinical point of view.[5]

In case of nasal augmentation the autogenous material is the best option and has given far superior results.[6,7] The silicone implant tends to be "mobile" and the HDPE implant does not look and feel good. In every given scenario, the first choice should be an autogenous bone and only when it is not practical should alloplastic material be considered. One should not be carried away by this fad of using one or other alloplastic implant that is "custom-made" and can be done with "minimal fuss". We should explain to the patient the "real picture" of alloplastic material. Of course there can be situations where one has no choice but to fall back upon the alloplastic material-paucity of autogenous material, unwillingness on the part of the patient or need to "finish of the surgery" quickly because of poor surgical risk.

In conclusion, this is a large series wherein the use of HDPE implants in facial reconstructions in the Indian scenario have been shown to be "satisfactory" provided certain precautions are taken regarding the site, size and route of implant insertion. In carefully selected group of patients, HDPE implants can be an alternative to autogenous material. We need to discuss the potential complications and limitations with the patients. However, autogenous material should always remain the first choice for contour corrections/augmentations in the facial region.
